# Collateral Vein Ligation for Arteriovenous Fistula Maturation: A Pilot Study

**DOI:** 10.1016/j.ejvsvf.2025.03.001

**Published:** 2025-03-08

**Authors:** Bogdan Bratu, Bettina Chenesseau, Andreea Luchianov, Salomé Kuntz, Nabil Chakfé, Anne Lejay

**Affiliations:** a Department of Vascular Surgery, Hôpital Emile Muller, Mulhouse, France; b Vascular Surgery, Clinique du Diaconat, Mulhouse, France; c Deparment of Nephrology, Hôpital Emile Muller, Mulhouse, France; d Department of Vascular Surgery and Kidney Transplantation, University Hospital of Strasbourg, France

## Abstract

**Objectives:**

Native arteriovenous fistulae (AVF) may fail to achieve adequate blood flow or size for successful cannulation and dialysis. No clear strategy exists concerning the effectiveness of collateral vein ligation (CVL) to improve AVF maturation. The aim of this study was to evaluate the effectiveness of CVL in improving AVF maturation.

**Methods:**

A retrospective study was performed, including all patients who underwent CVL for delayed AVF maturation between January 2023 and December 2023. Combined procedures, such as concomitant venous stenosis angioplasties, were excluded. Evolution of AVF flow after CVL compared with AVF flow before CVL was recorded. The primary endpoint was defined as successful maturation after CVL. The AVF was considered mature when it could be routinely cannulated for the total duration of dialysis, for at least six months.

**Results:**

Median follow up was eleven months (range 6–14 months). CVL allowed successful maturation in five of the six patients, with a median AVF flow increase of 44%. In these five patients, sustained dialysis after CVL was uneventful, without need for any additional interventions.

**Conclusion:**

These results highlight the potential effectiveness of CVL in improving AVF maturation, although larger studies are needed to confirm these findings.

## HIGHLIGHTS

• Collateral vein ligation can improve arteriovenous fistula maturation.
• Selection of patients is necessary.
• Collateral vein diameter at least 60% of the draining vein help selecting patients.

## INTRODUCTION

The creation of an arteriovenous fistula (AVF) immediately increases blood flow through the vein due to the pressure gradient created. AVF maturation depends on several changes involving the vein such as increased blood flow, increased vein diameter, and increased vein visibility. Successful AVF creation results in early cannulation and adequate blood flow to support dialysis. An adequate AVF resides around 0.6 cm from the skin surface, has a flow >600 mL/min, and a diameter >0.6 cm.^1^ However, 10–35% of AVFs fail to achieve adequate blood flow or size for successful cannulation.^2^, ^3^, ^4^ All AVFs should be routinely evaluated after between four to six weeks when normal post-operative oedema will have resolved. It is then expected that the majority of AVFs will have reached maximum flow.^1^ If there is evidence of dysfunction, early intervention is advocated to identify and solve the underlying problem.

Venous outflow lesions are the most common lesions found in non-maturing AVF. Such lesions may be due to pre-existing anomalies, such as small calibre veins, fibrotic or stenotic veins, or sites of previous puncture or catheter placements. In addition, venous outflow problems may be due to collateral veins that limit proper AVF development, since collateral veins can divert flow away from the downstream aspect of the AVF. Collateral veins can steal sufficient pressure and flow to decrease vein wall remodelling and AVF calibre enlargement. Collateral veins can be any branch vein arising anywhere from the arteriovenous anastomosis to the most central aspect of the planned puncturable portion of the AVF. There are often several collateral veins, but not all will be diverting flow due to functional valves or small size. A collateral vein of at least 20% of the diameter of the upstream draining vein can be considered as a risk of non-maturation.^5^

While the treatment of stenosis is well established with clear strategies, the management of collateral veins lacks well defined scientific evidence, and no recommendation is provided in the Clinical Practice Guidelines of the European Society for Vascular Surgery.^1^ The main objective of this pilot study was to evaluate the effectiveness of collateral vein ligation (CVL) in improving AVF maturation.

## MATERIALS AND METHODS

### Inclusion and exclusion criteria

A retrospective study including all patients who underwent CVL for delayed AVF maturation between January 2023 and December 2023 was performed. Combined procedures, such as concomitant venous stenosis angioplasties, were excluded. The study was conducted in accordance with the local legislation and institutional requirements. Written informed consent was obtained from all patients in accordance with the national legislation and institutional requirements.

### Demographic parameters

Demographics (sex, age), cardiovascular risk factors (hypertension, dyslipidaemia, diabetes, obesity), and cause of chronic kidney disease of the patients were recorded.

### Duplex ultrasound parameters

Pre-operative duplex ultrasound (DUS) data, including artery and vein diameters before AVF creation, were recorded for all patients. DUS was performed six weeks after AVF creation. AVF flow rate, draining vein diameter, and depth were recorded. It was considered that an adequate AVF for dialysis, according to the Kidney Dialysis Outcome Quality Initiative guidelines, should have a flow rate of at least 600 mL/min, a draining vein diameter of at least 0.6 cm, and a depth of less than 0.6 cm from the surface of the skin.^1^^,^^2^ The collateral vein diameter was also recorded and the ratio between collateral vein diameter and draining vein diameter was calculated.

DUS was performed six weeks after CVL. Change of AVF flow after CVL compared with AVF flow before CVL was recorded.

### Outcomes

The primary endpoint was defined as successful maturation after CVL. AVF was considered mature when it could be routinely cannulated for the total duration of dialysis, for at least six months. Secondary endpoints were increase in AVF flow rate and time between CVL and dialysis.

## RESULTS

During the study period, 165 AVF were created. Among these, six patients underwent CVL for AVF delayed maturation (four men and two women). The median age was sixty-six years (range 49–70). All patients had hypertension and dyslipidaemia, four patients were diabetic, and one patient was obese. The reason for chronic kidney disease was diabetic nephropathy in four cases, polycystic kidney disease in one case, and single functioning kidney in one case.

### Duplex ultrasound parameters before arteriovenous fistula creation

According to pre-operative DUS parameters, radiocephalic access was selected in all six cases. Median radial artery diameter was 2 mm (range 1.9–2.5) and median cephalic vein diameter 3 mm (range 2.1–4.2). Left radiocephalic access was performed in four cases and right radiocephalic access in two cases.

### Duplex ultrasound parameters after arteriovenous fistula creation

After AVF creation, median AVF flow rate was 547 mL/min (range 380–625), median draining vein diameter was 5.5 mm (range 5–7.3), and median depth of the draining vein was 5 mm (range 3–6). The median collateral vein diameter was 3.5 mm (range 2.8–5.6) and median ratio was 62% (range 56–77).

### Collateral ligation

CVL was performed under local anaesthesia in the operating room. The surgical procedure was uneventful in all patients.

### Duplex ultrasound parameters after collateral vein ligation

After CVL, median AVF flow rate was 720 mL/min (range 500–975), and the median AVF flow increase was 44% (range 0–71).

### Outcomes

After CVL, maturation was obtained, and dialysis could be initiated in five of the six patients (83%). Table 1 summarises DUS parameters and outcomes of the patients.

**Table 1:** Duplex ultrasound parameters after arteriovenous fistula (AVF) creation and after collateral ligation.

Case	Sex	Age	Post AVF creation						Post collateral ligation		
			Flow rate – mL/min	DV diameter – mm	DV depth – mm	CV diameter – mm	Ratio CV/DV – %	Dialysis	Flow rate – mL/min	Flow increase – %	Dialysis
1	M	65	400	7.3	5	5.6	77	No	500	25	Yes
2	F	65	595	6	4	4	67	No	790	33	Yes
3	M	49	598	5	6	2.8	56	No	600	0	No
4	M	67	380	5	3	3	60	No	650	71	Yes
5	F	68	500	5.9	5	4	68	No	800	60	Yes
6	M	70	625	6	5	3.7	62	No	975	56	Yes

AVF = after arteriovenous fistula; DV = draining vein; CV = collateral vein.

The median interval between CVL and dialysis in these five patients was three months (range 2–6). For these five patients, sustained dialysis was possible and follow up was uneventful (median follow up eleven months, range 6–14). No additional intervention was required.

In one patient CVL did not achieve maturation. In this patient, the draining and collateral vein diameters were 6 and 2.8 mm, respectively. The ratio between collateral vein and draining vein diameters was 56%. AVF flow before and after CVL was unchanged (596 and 600 mL/min respectively). Phlebography was performed and showed a central vein stenosis. Angioplasty was performed, allowing later cannulation (AVF flow was 750 mL/min).

## DISCUSSION

In this pilot study, CVL allowed dialysis initiation in five of the six patients, with a median AVF flow increase of 44%. These results highlight the potential effectiveness of CVL in improving AVF maturation, although additional studies with more robust sample sizes are needed to confirm these findings. Moreover, objective data are needed in order to select the patients in whom CVL might be effective.

CVL has been poorly investigated, and the literature is scarce and heterogeneous. CVL is rarely performed, and, when performed, different approaches are used (coil embolisation, surgical ligation, or percutaneous non-surgical ligation) for different indications (delayed maturation or distal ischaemia).^6^, ^7^, ^8^, ^9^, ^10^ Moreover, combined procedures (mostly including draining vein angioplasty) are often performed. In a retrospective review performed on 145 non-maturing AVFs, management of a collateral vein was reported in 12 cases, using coil embolisation, and allowed maturation in ten cases (83%).^7^ In another study, management of collateral veins was reported in 17 patients, using percutaneous ligation, and allowed maturation in 15 patients (88%).^8^ In another larger study, 187 patients with a poorly maturing AVF related to branch diversion (although combined with other interventions to achieve maturation) were analysed: 81 were treated with coil embolisation and 106 had CVL. Technical success was 90% for endovascular treatment and 100% for open treatment. Successful cannulation was obtained in 61% after endovascular treatment and 64% after open treatment.^10^ However, outcome comparisons between different approaches is difficult, due to the heterogeneity among indications, populations, and combined procedures. Accordingly, the ideal technique for CVL is not defined, but outcomes between different approaches seem comparable.

In most of the studies, collateral vein assessment was performed with angiography.^7^^,^^8^ However, DUS can be considered a key tool for assessing fistula maturation, measuring both AVF flow before and after CVL, as well as changes in the main draining vein. In this pilot study, pre- and post-ligation measurements objectively demonstrated improved draining vein calibre and increase of AVF flow, which are positive indicators of maturation.

From a physiological point of view, the beneficial effects of CVL can be attributed to vascular remodelling.^11^ The primary goal of CVL is to redirect blood flow to the main vein, significantly increasing flow in the latter. In fact, the collateral vein disperses blood flow and limits pressure in the main vein. CVL concentrates the flow toward the primary draining vein. This increased flow is essential for proper fistula maturation since it generates higher wall stress in the main vein.^12^ It has been postulated that this stress allows remodelling of the venous wall, making it more resilient and arterialised. It has also been assumed that the presence of collateral veins can create blood flow turbulence in the main vein, favouring the development of later stenosis. CVL could therefore theoretically reduce this risk, but this has not been tested in a clinical situation.

However, the lack of evidence supporting CVL effectiveness in robust sample size studies, as well as homogeneity between reported techniques make it difficult to provide objective criteria to select patients in whom CVL would be effective.^13^ Some authors rely on visual assessment of flow through the collateral vein and its diameter, considered significant if greater than around one third of the draining vein diameter.^14^ In other studies, accessory vein size is not correlated with maturation rate.^7^^,^^8^ This discrepancy explains why computational fluid dynamics simulations were performed to analyse blood flow in AVFs.^15^ It has therefore been shown that the neither location nor collateral vein take off angle altered flow. When the diameter of the collateral vein was 33% of the diameter of the draining vein, flow in the collateral vein was 7% of the flow of the draining vein, but when the diameter of the collateral vein was increased to 50% and 66% of the diameter of the draining vein, flow through the collateral vein was 10% and 31%, respectively, of the flow in the main draining vein. Based on such computational fluids studies, it has therefore been suggested that CVL should be considered (1) when collateral vein diameter is at least 60% that of the draining vein, (2) when collateral vein diameter is 50% that of the draining vein with at least one other collateral exceeding 40% in diameter, or (3) when the collateral vein diameter is 50% that of the draining vein but divides into branches of the same size.^15^ In this pilot study the diameters of the collateral vein and the draining vein were assessed by DUS. It seemed that a collateral vein diameter at least 60% that of the draining vein could be considered as a threshold for maturation after CVL, supporting previous findings.

In conclusion, this pilot study provides information on the criteria for CVL. However, the sample size is too small to draw definitive conclusions and larger studies are needed. A larger prospective study including a control group is in progress. Moreover, initial results are encouraging, but long term follow up is missing. Long term data would be essential to evaluate the outcome of the procedure. However, this is real life experience and might help clinicians in current practice.

## FUNDING

None.

## CONFLICTS OF INTEREST

None.
